# A Proposal for Nomenclature in Myeloid C-Type Lectin Receptors

**DOI:** 10.3389/fimmu.2019.02098

**Published:** 2019-09-06

**Authors:** Carlos del Fresno, Francisco J. Cueto, David Sancho

**Affiliations:** Immunobiology Lab, Centro Nacional de Investigaciones Cardiovasculares (CNIC), Madrid, Spain

**Keywords:** lectin receptors, signaling, monocytes, macrophages, dendritic cells, innate immunity, nomenclature

## Abstract

Myeloid C-type lectin receptors (CLRs) comprise a family of receptors expressed by immune myeloid cells that share homologous C-type lectin domains. The implication of these CLRs in the regulation of homeostasis and activation of myeloid cells has generated a buoyant growth in the number of studies involving these receptors. Since their first description, diverse nomenclature has been used to refer to each of them, ranging from systematic classifications, such as gene name or cluster of differentiation, to non-systematic ones that include terminology based on gene expression patterns or function. In this review, we aim to summarize the different names used for the main myeloid CLRs and analyze which of them have been more frequently used in the literature. In addition, we have examined the evolution of the terminology applied to these myeloid CLRs over time. Based on this analysis, we propose a *consensus alias* for each of those myeloid CLRs. However, we acknowledge that systematicity is required beyond this terminology based on use frequency. Therefore, we have included gene names as the standardization tool to gather the maximum agreement. We suggest that a standard nomenclature consisting of both gene names and consensus alias should be included at least in scientific abstracts, which would help to identify relevant literature, saving time and effort and fostering the research in this field in a more systematic manner.

## Broad Diversity of Myeloid C-Type Lectin Receptors and Babylonian Confusion in Their Naming

Innate immune cells surveil their nearby microenvironment, reacting against different challenges when they are identified. This reaction is based in a toolbox consisting of a plethora of pattern recognition receptors (PRRs) capable of sensing both pathogen-associated molecular patterns (PAMPs) present in invading microbes (1) and damage-associated molecular patterns (DAMPs), which are molecules released by stressed or necrotic cells (2). Ligation of PRRs by their ligands initiates intracellular signaling pathways that modulate innate and adaptive immune responses. Among the described PRRs, we will focus here in C-type lectin receptors (CLRs) expressed by myeloid cells such as monocytes, macrophages, or dendritic cells.

C-type lectin receptors (CLRs) comprise a large family of metazoan proteins (more than 1,000) characterized by containing at least one C-Type Lectin-like Domain (CTLD) (3). In order to organize the large number of receptors included in this superfamily, diverse classifications have been proposed. CLRs were early classified into 17 groups based on their structure (3). As the functional relevance of this structural classification is limited, an alternative classification of myeloid transmembrane CLRs was proposed based on their intracellular signaling domains, namely Immunoreceptor Tyrosine-based Activation Motif (ITAM) domains, hemITAM domains, Immunoreceptor Tyrosine-based Inhibition Motif (ITIM) domains or CLRs without clear ITAM or ITIM domains (4, 5).

Moreover, the abundance and diversity in function and expression of myeloid CLRs has also contributed to a great dispersion in the way that researchers name these receptors. These myeloid CLRs were initially named based on their expression pattern or gene location (e.g., DEndritic cell-associated C-type lecTIN-1 for Dectin-1 (6), Dendritic cell NK lectin Group Receptor-1 for DNGR-1 (7), Macrophage-INducible C-type LEctin for MINCLE (8). Later on, a serial nomenclature was adopted for the naming of genes from the CLR family, based on their common domain, so that they are all cataloged as CLEC (C-type LECtin) followed by an alphanumerical identifier (9). In an attempt to avoid confusion when identifying surface markers, Human Leukocyte Differentiation Antigen Workshops were organized to standardize the naming of markers that are recognized by specific monoclonal antibodies. The result of this effort was the definition of the cluster of differentiation (CD) nomenclature (10), which also applies to CLRs, such as CD303 for *CLEC4C*/BDCA-2 (11). The last of these workshops hold in 2014 represented the tenth of these events and provided CD nomenclature for some more CLRs, reaching the CD371 for CLEC12A (12).

This diverse nomenclature used for myeloid CLRs may generate confusion when searching or disseminating information as illustrated for CLRs expressed on dendritic cells, where up to seven different names can be found for some of these receptors (13). In an attempt to systematically analyze potential solutions to this Babylonian confusion in the myeloid CLR field, we have herein listed the different names used to identify myeloid CLRs at the National Center for Biotechnology Information (NCBI) database (https://www.ncbi.nlm.nih.gov). We have studied the prevalence of each of these names and the evolution of their usage over time in title, abstract, and keywords of published works. We decided to delimit the survey to these sections of the manuscripts as they are the main sections where scientists search literature of their interest using the PubMed search tool from NCBI (14). Taking into account this information, we propose a standard nomenclature consisting of a *consensus alias* for each myeloid CLR based on the most frequently used in the current literature. In any case, our study illustrates the need for systematization in the naming of myeloid CLRs, and thus we propose that the gene name (based on the CLEC nomenclature) should always accompany the “consensus” identifier at least in the abstract. Importantly, the official nomenclature for naming of human genes is provided by the Human Genome Nomenclature Committee (HGNC) (www.genenames.org), while official mouse gene names come from the Mouse Genome Informatics (MGI) (www.informatics.jax.org).

## Analysis of Names Used for Myeloid CLRs

**Figure 1:**
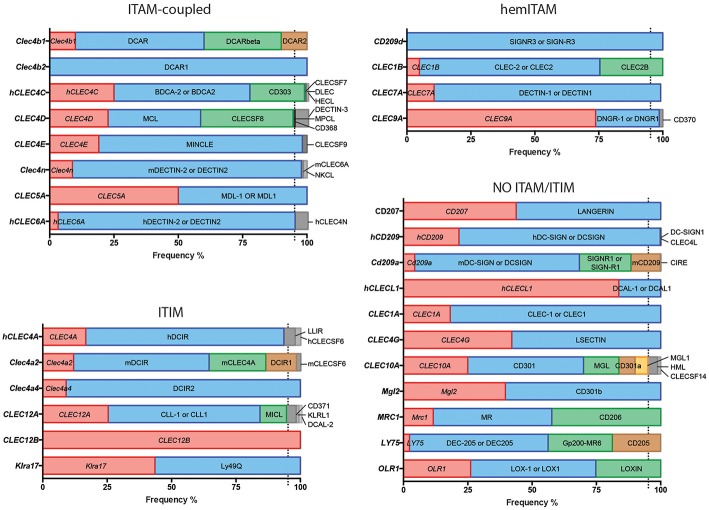
Usage frequencies of the different aliases provided by NCBI for every myeloid C-type lectin receptor surveyed in this review. Receptors are grouped based on their intracellular domain and listed in alphabetical order of their gene name. Colored bars represent names with usage frequencies higher than 5%, except for gene names, which are always in red regardless their prevalence.

In order to study the use of the different denominations, we selected a list of myeloid CLRs grounded on our former review on the flexibility of these sensors to trigger different signaling pathways (5). Based on the “Gene” resource of NCBI (https://www.ncbi.nlm.nih.gov/gene/) we listed all the potential names (aliases) used for the mouse and human version of each selected receptor, performing the search based on their gene names, and organizing the CLRs based on their functional intracellular domains as previously proposed (4, 5) (Figure 1).

Next, we completed a search based on the PubMed resource of NCBI (https://www.ncbi.nlm.nih.gov/pubmed/, performed along the second and third week of March 2019) for any of the provided aliases for each receptor. The scope of this survey was to obtain the total number of references where each specific name has been used either alone or combined with other aliases for the same CLR, generating usage frequencies for each of these terms (Figure 1). Colored bars represent names used in more than 5% of total articles referring to that CLR, except for gene names as a source of systematicity, which are always depicted in red independently of their frequency. An additional layer of confusion for naming myeloid CLRs relies on the use or not of hyphens in their names. For our study, those aliases found in both versions were clustered as a single search using the “OR” command (Figure 1).

A quick view clearly illustrates the variability of alias type across CLRs. It can range from receptors always appointed with the same alias, either their gene (*CLEC12B*) or alternative (DCAR1) name, to members identified with up to five different aliases in frequencies over 5% such as *CLEC10A*, CD301, MGL, CD301a, and MGL1 (and even found named as HML or CLECSF14 in minor proportions). This dispersion occurs regardless the classification based on intracellular domains.

This analysis highlights the need for a systematic nomenclature for myeloid CLRs. This is particularly important for those receptors expressed by diverse coding genes in different species, but commonly found with a shared alias. This is the case of ITIM-bearing DCIR, encoded by *CLEC4A* in humans and by *Clec4a2* in mice. In both cases, DCIR is one of the accepted aliases and is thus an unspecific name. Contrarily, and still using DCIR as an example, some names exclusively designate the human (LLIR) or mouse (DCIR1) receptors, but these aliases are not among the most frequent. Therefore, for our study, we have distinguished between “human DCIR” and “mouse DCIR,” as they are encoded by genes with a different name. These same criteria were applied to an additional name for DCIR, CLECSF6, also shared for human and mouse receptors. In addition, mouse DCIR is linked to several isoforms, mainly those encoded by the genes *Clec4a2* and *Clec4a4* and literature on these isoforms may be confusing if the genes are not named.

The use of non-official aliases, even not included at the NCBI gene database, constitutes another anomalous situation. This is the case for the non-ITAM/ITIM coupled CD207, commonly known as LANGERIN, with CD207 used in 43,82% of references in PubMed and LANGERIN in the remaining 56,17%. Taking this into account, for this receptor in particular, we have performed our analysis including both names, even the one not recorded as a gene alias at the Gene tool (LANGERIN). The name LANGERIN comes from the specific expression of this CLR by Langerhans cells. These cells show unique intracellular structures called Birbeck granules whose presence is associated with LANGERIN/CD207 expression (15). Therefore, this CLR can be found both intracellularly and extracellularly (16). Taking this into account, we wondered whether the use of LANGERIN or CD207 correlates with the detection of the receptor by histologic methods often used for intracellular staining or flow cytometry, usually more associated to extracellular detection. To approach it, we crossed in Pubmed either LANGERIN or CD207 with “histology” or “flow cytometry.” Interestingly, both aliases were more frequently found associated to histology (84,6% for LANGERIN and 82,86% for CD207). The same happened when the search was performed combining LANGERIN AND CD207 (84.7%). This would mean that histologic techniques are preferred for studies involving this CLR and that both names are used indistinctly.

A combination of these circumstances occurs for the ITAM-coupled DECTIN-2. According to the Gene resource, DECTIN-2 or DECTIN2 refers only to the product of the human *CLEC6A* gene but not to the mouse *Clec4n*. However, it is quite common to find references in the literature quoting DECTIN-2 for the mouse version, which can be specifically found as NKCL. Still, CLEC4N is also recorded as an alternative name for the human version. Therefore, we have differentiated between “human DECTIN-2 or DECTIN2” and “mouse DECTIN-2 or DECTIN2,” applying the same criteria to CLEC4N and CLEC6A.

Another representative example of the divergence in mouse and human terms for a myeloid CLR is DC-SIGN, a member of these receptors not coupled to an identifiable ITAM/ITIM domain. The genes encoding for this receptor are named differently in human (*CD209*) and mouse (*Cd209a*). DC-SIGN/DCSIGN can be widely found referring to both forms, consequently, we have also differentiated between “human DC-SIGN or human DCSIGN” and “mouse DC-SIGN or mouse DCSIGN.” In this case, the mouse receptor is specifically identified as SIGNR1 or SIGN-R1, but CD209 is also considered an alias for the mouse version. Therefore, we have differentiated between human and mouse CD209 for our analysis. In any case, to some extent, we assume some overlapping on the results generated by these searches based on “human” or “mouse.” It is interesting to see how this CLR was initially described as a membrane-associated mannose-binding receptor for the HIV gp120 protein (17), with no specific name until it was first identified as DC-SIGN (18). Therefore, our analysis applies to the use of different nomenclatures for CLRs, although biological information about some of them could have been generated before their current names were coined.

It is also worthy to comment that the frequency of use of a name can be influenced by the contribution of a particular author to the study of a CLR, specially when the literature is not abundant. Therefore, the most frequently used nomenclature may be biased by the publishing frequency of one author on a particular CLR. This notion emphasizes the need for an agreement in the way of naming myeloid CLRs.

Indeed, in light of the results shown in Figure 1, it is clear that defining a systematic manner to refer to myeloid CLRs is mandatory. In that sense, we encourage that, independently of how we prefer naming our favorite myeloid CLR, we should all include the gene name (as listed in Figure 1) to unequivocally and systematically identify the receptor, both in the abstract and the first time that they are mentioned in the text.

## Nomenclature Evolution of Myeloid C-Type Lectin Receptors

**Figure 2:**
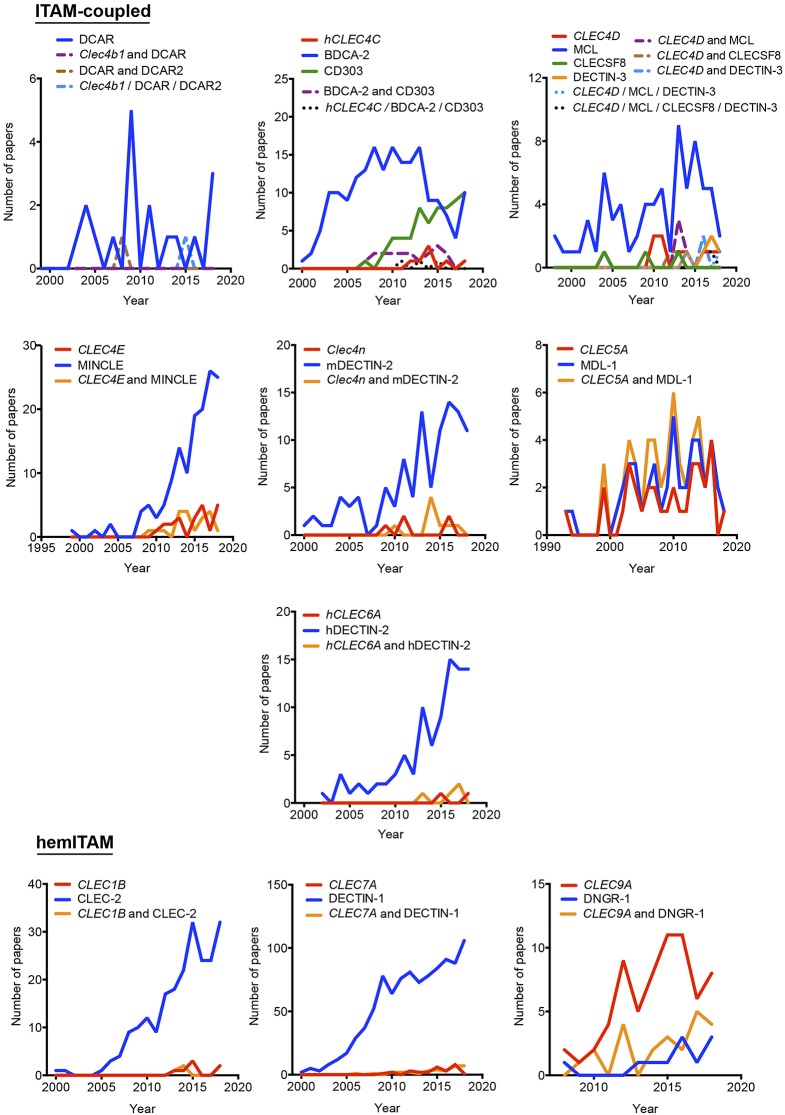
Temporal analysis of the use of different aliases for ITAM- and hemITAM-coupled myeloid C-type lectin receptors. The frequency of each name was analyzed either alone or in combination with the alternative aliases.

**Figure 3:**
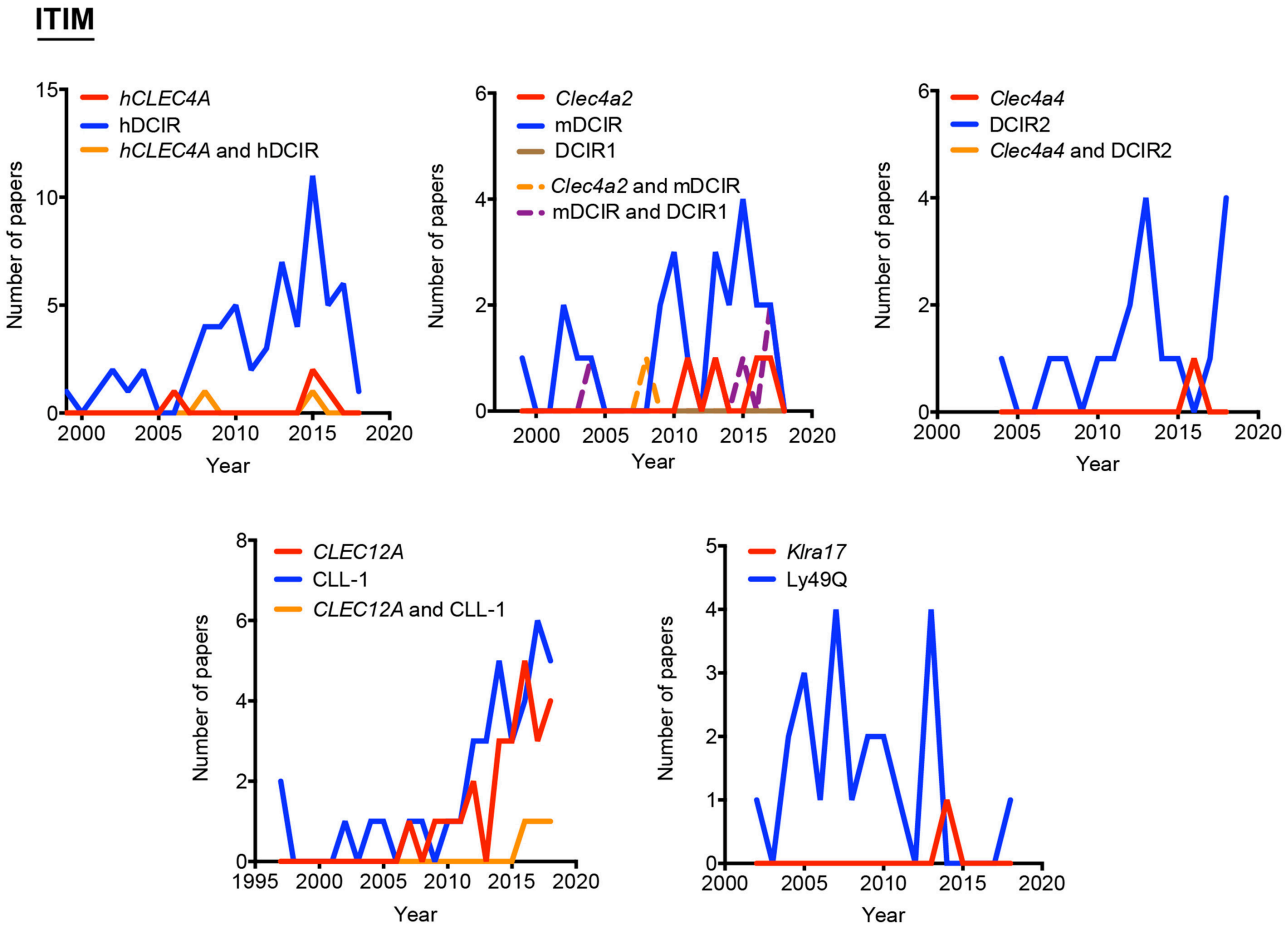
Use of different names along time for myeloid C-type lectin receptors bearing an ITIM motif in their intracellular domain. The frequency of each alias was analyzed either alone or in combination with their alternatives.

**Figure 4:**
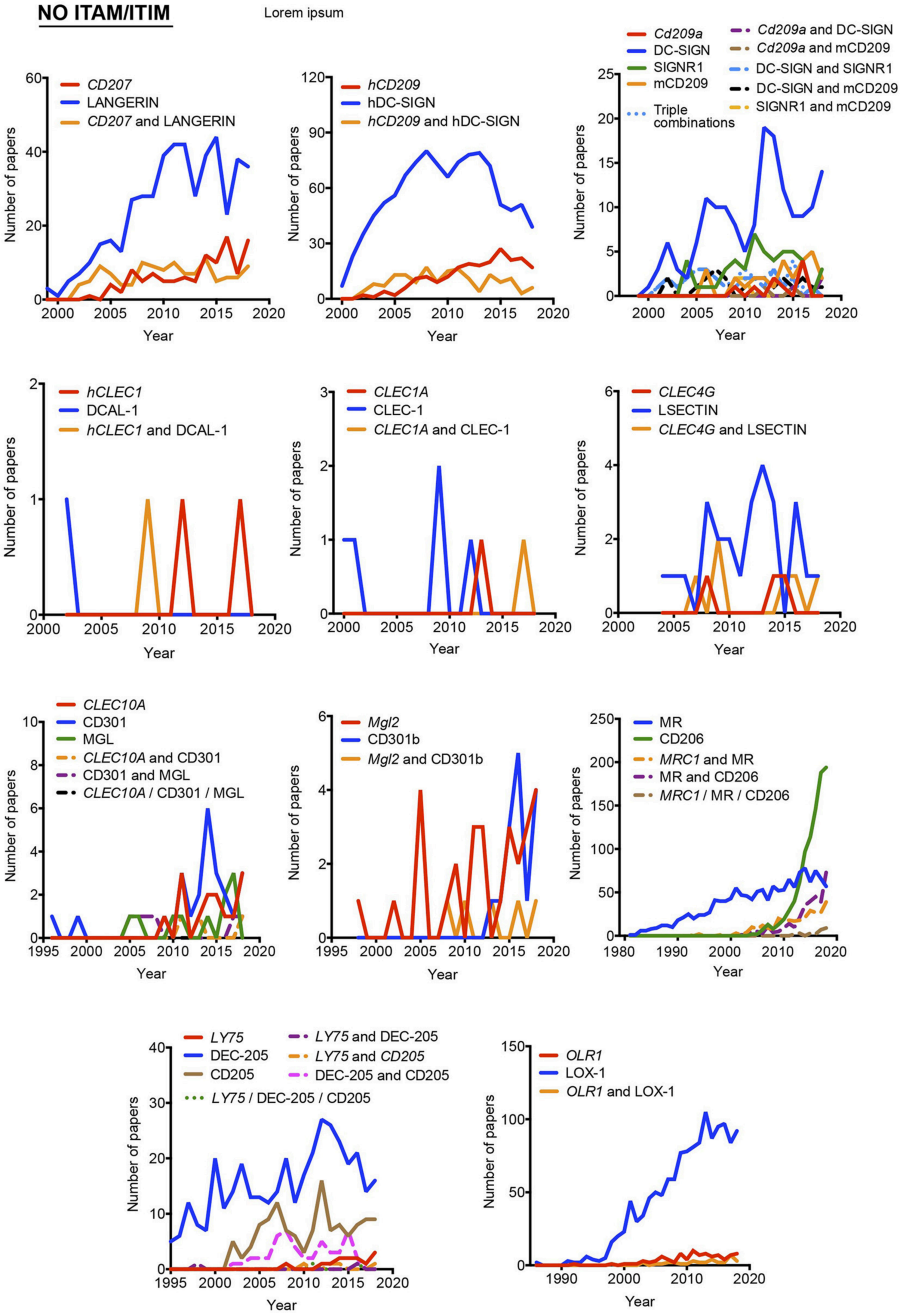
Usage evolution of different aliases for C-type lectin receptors not coupled to an identifiable ITAM or ITIM motif in their intracellular domain. The frequency of each name was analyzed either alone or in combination with the alternative aliases.

Our proposal assumes that there are some common and traditionally used nomenclatures that do not include the systematicity provided by the gene name. To define the temporal evolution of preferred names for myeloid CLRs, we analyzed the usage of these names (included in Figure 1) in the last years, which could suggest a consensus alias for each myeloid CLR. We analyzed the number of works naming a particular term either in the title, abstract, or keywords of published works. The search results were exported and a table including complete title, abstract, and keywords for each of the found items was generated using the reference manager software JabRef (www.jabref.org). Note that for each CLR, we incorporated the “NOT” command to search for literature where each specific alias had been used exclusively, but not the other aliases. We determined the use of each alias in any of these three sections for every paper. For the sake of clarity and unless it applies to gene names, we only analyzed aliases with frequencies over 5% for timeline analysis. Next, by using the “AND” command, we analyzed the number of simultaneous appearances among the selected aliases, in order to study their combined usage. Results are shown in Figure 2 for ITAM- or hemITAM-coupled myeloid CLRs, Figure 3 for ITIM-bearing and Figure 4 for those receptors that do not bear any ITAM or ITIM domain. Those receptors with a single alias in Figure 1 have not been included in this study.

Timeline analysis may not completely match the results included in Figure 1; Jabref exploits the Machine readable digital library (Mr. DLib) for their searches while PubMed search engine is based on the Medical subjet headings (MeSH) algorithm (19). This fact could introduce some variability. On the other hand, the analysis performed in Figure 1 did not include the “NOT” command; therefore, those references where a myeloid CLR is identified by two (or more) aliases, were included in the total number accounting for both names. However, in the temporal analysis, those examples will be only included in the combinations, identified by the use of the “AND” command. The CLR devoid of ITAM/ITIM motif *MRC1* is a representative example, where all quotations in the temporal analysis using this identification are combined with other aliases. This would also suggest the inclusion of a consensus alias at least, in the abstract of manuscripts, because if we consider the growing amount of literature that we need to handle, representative abstracts are becoming fundamental for the selection of relevant papers in scientific research.

As indicated above, hyphens have been used almost randomly in some aliases to separate name and numbers. Both versions are documented in Figure 1 as, for example, BDCA-2 or BDCA2. For this reason, we included both styles in the temporal analysis, and only the most prevalent version is depicted in Figures 2–4. This study illustrates that for all the instances except SIGNR3/SIGN-R3 and SIGNR1/SIGN-R1, the version including hyphen is the predominant one and consequently, we encourage its use. The limitation in the number of characters accepted in certain journals might be behind the use of names lacking the hyphen. However, authors should adhere to consensus aliases to make their research more easily identifiable.

The temporal analysis illustrates that common names have been more frequently used than gene names as a systematic manner to identify myeloid CLRs. *CLEC9A* vs. DNGR-1 represent the only exception of a CLR more often identified by its gene name. The use of *CLEC9A* is extended as a gene marker for cDC1s (20, 21), while the use of DNGR-1 is linked to functional studies on this receptor (22, 23). *CLEC5A* shows a peculiar behavior, with virtually the same frequency for exclusive use of either the gene name or MDL-1 or the combination of both of them.

In general, gene names and main aliases are not combined together in the abstract, as we propose, which contributes to confusion. Thus, as indicated before, the same common name (DC-SIGN) has been applied to the mouse and human versions without distinction, although they are encoded by different genes and show different expression patterns (24).

Interestingly, the study of the temporal evolution also allows for the detection of preferred names for CLRs along the time, which could be an extra criterium to propose a consensus alias. In this line, it is remarkable the use of the cluster of differentiation nomenclature for some receptors such as *CLEC4C*/CD303, *CLEC10A*/CD301, *Mgl2*/CD301b, *MRC1*/CD206, and *LY75*/CD205. Once defined in a HLDA workshop, their frequencies overcame any of their other aliases. The best example is CD206 for the mannose receptor *MRC1*, used both alone and in combination with other aliases. This is because the first reference to a CD occurs after monoclonal antibodies are submitted to a HLDA workshop and, from there onwards, the use of the CD nomenclature for a specific receptor begins to be applied for multiple applications (25).

## Proposal of Consensus Nomenclature for Myeloid CLRs

Considering all our analysis, Table 1 compiles our proposal for the consensus alias that should be used when referring to any myeloid CLRs surveyed in this review. This proposal is based both in the total frequency (Figure 1) and temporal evolution (Figures 2–4) of their usage. In order to introduce systematicity, we propose that the current most frequent alias should be always accompanied by the specific gene name. Furthermore, we encourage the use of hyphens when required, as they are more frequently used.

**Table 1:** Proposed consensus alias for myeloid C-type lectin receptors.

**ITAM-COUPLED**	
***Clec4b1***	**DCAR**
***Clec4b2***	**DCAR1**
***CLEC4C***	**CD303**
***CLEC4D***	**MCL**
***CLEC4E***	**MINCLE**
***Clec4n***	**mDECTIN-2**
***CLEC5A***	**MDL-1**
***CLEC6A***	**hDECTIN-2**
**ITIM**	
***CLEC4A***	**hDCIR**
***Clec4a2***	**mDCIR**
***Clec4a4***	**DCIR2**
***CLEC12A***	**CLL-1**
***CLEC12B***	**CLEC12B**
***Klra17***	**Ly49Q**
**hemITAM**	
***CD209d***	**SIGNR3**
***CLEC1B***	**CLEC-2**
***CLEC7A***	**DECTIN-1**
***CLEC9A***	**DNGR-1**
**NO ITAM/ITIM**	
***CD207***	**LANGERIN**
***CD209***	**hDC-SIGN**
***CD209a***	**mDC-SIGN**
***CLECL1***	**DCAL-1**
***CLEC1A***	**CLEC-1**
***CLEC4G***	**LSECTIN**
***CLEC10A***	**CD301**
***Mgl2***	**CD301b**
***MCR1***	**CD206**
***LY75***	**DEC-205**
***OLR1***	**LOX-1**

*To facilitate identification, both gene name and alias should be named in the abstract of the manuscript*.

Both the gene name and the proposed consensus alias (Table 1) should appear at least in the abstract of manuscripts or meetings. This practice would facilitate the identification of literature of interest, fostering the visibility of any work in their research field.

## Author Contributions

CF, FC, and DS: conceptualization. CF and FC: methodology, analysis, and investigation. CF: writing–original draft and preparation of figures. FC and DS: writing–review and editing. CF and DS: funding acquisition and supervision.

### Conflict of Interest Statement

The authors declare that the research was conducted in the absence of any commercial or financial relationships that could be construed as a potential conflict of interest.

## References

[1] Janeway CA. Approaching the asymptote? Evolution and revolution in immunology. Cold Spring Harb Symp Quant Biol. (1989) 54(Pt 1):1–13. 10.1101/SQB.1989.054.01.003 2700931

[2] Matzinger P. The danger model: a renewed sense of self. Science. (2002) 296:301–5. 10.1126/science.1071059 11951032

[3] Zelensky AN Gready JE. The C-type lectin-like domain superfamily. FEBS J. (2005) 272:6179–217. 10.1111/j.1742-4658.2005.05031.x 16336259

[4] Sancho D Reis e Sousa C. Signaling by myeloid C-type lectin receptors in immunity and homeostasis. Annu Rev Immunol. (2012) 30:491–529. 10.1146/annurev-immunol-031210-101352 22224766 PMC4480235

[5] del Fresno C Iborra S Saz-Leal P Martínez-López M Sancho D. Flexible signaling of myeloid C-type lectin receptors in immunity and inflammation. Front Immunol. (2018) 9:804. 10.3389/fimmu.2018.00804 29755458 PMC5932189

[6] Ariizumi K Shen GL Shikano S Xu S Ritter R Kumamoto T. Identification of a novel, dendritic cell-associated molecule, dectin-1, by subtractive cDNA cloning. J Biol Chem. (2000) 275:20157–67. 10.1074/jbc.M909512199 10779524

[7] Sancho D Mourão-sá D Joffre OP Schulz O Rogers NC Pennington DJ. Tumor therapy in mice via antigen targeting to a novel, DC-restricted C-type lectin. J Clin Invest. (2008) 118:2098–110. 10.1172/JCI34584DS1 18497879 PMC2391066

[8] Matsumoto M Tanaka T Kaisho T Sanjo H Copeland NG Gilbert DJ. A novel LPS-inducible C-type lectin is a transcriptional target of NF-IL6 in macrophages. J Immunol. (1999) 163:5039–48. 10528209

[9] Colonna M Samaridis J Angman L. Molecular characterization of two novel C-type lectin-like receptors, one of which is selectively expressed in human dendritic cells. Eur J Immunol. (2000) 30:697–704. 10.1002/1521-4141(200002)30:2<697::AID-IMMU697>3.0.CO;2-M 10671229

[10] Engel P Boumsell L Balderas R Bensussan A Gattei V Horejsi V. CD nomenclature 2015: human leukocyte differentiation antigen workshops as a driving force in immunology. J Immunol. (2015) 195:4555–63. 10.4049/jimmunol.1502033 26546687

[11] Röck J Schneider E Grün JR Grützkau A Küppers R Schmitz J. CD303 (BDCA-2) signals in plasmacytoid dendritic cells via a BCR-like signalosome involving Syk, Slp65 and PLCγ2. Eur J Immunol. (2007) 37:3564–75. 10.1002/eji.200737711 18022864

[12] Clark G Stockinger H Balderas R van Zelm MC Zola H Hart D. Nomenclature of CD molecules from the tenth human leucocyte differentiation antigen workshop. Clin Transl Immunol. (2016) 5:e57 10.1038/cti.2015.38 26900471 PMC4735060

[13] Kanazawa N. Dendritic cell immunoreceptors: C-type lectin receptors for pattern-recognition and signaling on antigen-presenting cells. J Dermatol Sci. (2007) 45:77–86. 10.1016/j.jdermsci.2006.09.001 17046204

[14] Delvin E Pillay TS Newman A How to write a scientific paper: practical guidelines. FEBS J. (2016) 283:3882–5. 10.1111/febs.13918 27870269

[15] Valladeau J Dezutter-Dambuyant C Saeland S. Langerin/CD207 sheds light on formation of birbeck granules and their possible function in langerhans cells. Immunol Res. (2003) 28:93–107. 10.1385/IR:28:2:93 14610287

[16] Mc Dermott R. Birbeck granules are subdomains of endosomal recycling compartment in human epidermal langerhans cells, which form where langerin accumulates. Mol Biol Cell. (2002) 13:317–35. 10.1091/mbc.01-06-0300 11809842 PMC65091

[17] Curtis BM Scharnowske S Watson AJ. Sequence and expression of a membrane-associated C-type lectin that exhibits CD4-independent binding of human immunodeficiency virus envelope glycoprotein gp120. Proc Natl Acad Sci USA. (1992) 89:8356–60. 10.1073/pnas.89.17.8356 1518869 PMC49917

[18] Geijtenbeek TB Kwon DS Torensma R van Vliet SJ F van Duijnhoven GC Middel J. DC-SIGN, a dendritic cell-specific HIV-1-binding protein that enhances trans-infection of T cells. Cell. (2000) 100:587–97. 10.1016/s0092-8674(00)80694-7 10721995

[19] Ecker ED Skelly AC. Conducting a winning literature search. Evid Based Spine Care J. (2010) 1:9–14. 10.1055/s-0028-1100887 23544018 PMC3609008

[20] Piva L Tetlak P Claser C Karjalainen K Renia L Ruedl C. Cutting edge: Clec9A+ dendritic cells mediate the development of experimental cerebral malaria. J Immunol. (2012) 189:1128–32. 10.4049/jimmunol.1201171 22732587

[21] Gilfillan CB Kuhn S Baey C Hyde EJ Yang J Ruedl C Clec9A + dendritic cells are not essential for antitumor CD8 + T cell responses induced by poly I:C immunotherapy. J Immunol. (2018) 200:2978–86. 10.4049/jimmunol.1701593 29507107

[22] del Fresno C Martínez-cano S Blanco-menéndez N Schulz O Gallizioli M DNGR-1 in dendritic cells limits tissue damage by dampening neutrophil recruitment. Science. (2018) 356:351–6. 10.1126/science.aan8423 30337411

[23] Zelenay S Keller AM Whitney PG Schraml BU Deddouche S Rogers NC. The dendritic cell receptor DNGR-1 controls endocytic handling of necrotic cell antigens to favor cross-priming of CTLs in virus-infected mice. J Clin Invest. (2012) 122:1615–27. 10.1172/JCI60644 22505458 PMC3336984

[24] Kalay H Unger WWJ García-Vallejo JJ van Kooyk Y Fehres CM. Glycan-based DC-SIGN targeting vaccines to enhance antigen cross-presentation. Mol Immunol. (2012) 55:143–5. 10.1016/j.molimm.2012.10.031 23158834

[25] Gez S Barber N Woolfson A Belov L Christopherson RI Mulligan SP. Profiling CD antigens on leukaemias with an antibody microarray. FEBS Lett. (2009) 583:1785–91. 10.1016/j.febslet.2009.03.018 19298816

